# Low syphilis treatment rates and associated birth outcomes in pregnant women with and without HIV in Zambia: A cohort study

**DOI:** 10.1002/ijgo.70518

**Published:** 2025-09-03

**Authors:** Albert Manasyan, Anna V. Jones, Yumo Xue, Herbert Kapesa, Maureen Mzumara, Jodie A. Dionne, Mwangelwa Mubiana‐Mbewe

**Affiliations:** ^1^ Department of Pediatrics, Division of Neonatology University of Alabama at Birmingham Birmingham Alabama USA; ^2^ Center for Infectious Disease Research in Zambia Lusaka Zambia; ^3^ Department of Obstetrics and Gynecology Washington University in St. Louis St. Louis Missouri USA; ^4^ Department of Medicine, Division of Infectious Diseases University of Alabama at Birmingham Birmingham Alabama USA

**Keywords:** antenatal care, benzathine penicillin G, HIV, pregnancy, syphilis, treatment

## Abstract

**Objective:**

Syphilis and HIV in pregnancy contribute to adverse birth outcomes in Africa. Benzathine penicillin G remains an effective treatment for syphilis in pregnancy, yet gaps persist in timely treatment. The aim of this study was to compare factors associated with adverse birth outcomes among pregnant women diagnosed with syphilis in health facilities according to their HIV status.

**Methods:**

This retrospective cohort analysis included pregnant women who screened positive for syphilis with routine rapid plasma reagin (RPR) testing in 10 antenatal care (ANC) clinics in Zambia between January 2018 and December 2019. Adverse birth outcomes (preterm delivery, low birth weight, fetal demise, congenital syphilis, and neonatal death) were collected through June 2020. Patient characteristics according to HIV status were compared using Pearson chi‐square test or Fisher exact test for categorical variables and Wilcoxon rank sum test for continuous variables. Logistic regression models were used to estimate the association between maternal and facility‐level factors and a composite measure of adverse birth outcomes.

**Results:**

In this cohort of 1204 pregnant women diagnosed with syphilis in health facilities, 42.5% had HIV coinfection and only 48.1% had documented penicillin treatment. Although preterm delivery rates were higher among women with syphilis and HIV (39.9% vs. 30.0% with syphilis alone; *P* = 0.003) the odds of having any adverse birth outcome were similar in both groups.

**Conclusion:**

Adverse birth outcomes were highly prevalent in Zambia among pregnant women with syphilis and treatment rates were low. Universal access to syphilis treatment in ANC clinic is needed to improve outcomes.

## 1 INTRODUCTION

Syphilis in pregnancy remains a critical global public health problem. In sub‐Saharan Africa (SSA), syphilis prevalence among women attending antenatal care (ANC) ranges from 0.1%–10%.^1^, ^2^ Despite the launch of the WHO Elimination of Mother‐to‐Child Transmission of Human Immunodeficiency Virus (HIV) and Syphilis (EMTCT+) initiative in 2014, antenatal syphilis screening and treatment rates in SSA remain low (49.0%), contributing to high rates of adverse outcomes such as low birth weight, preterm birth, stillbirth, fetal death, neonatal death, and congenital syphilis.^3^, ^4^, ^5^ More than 200 000 adverse birth outcomes in Africa each year are attributable to syphilis,^2^ yet infection is curable with benzathine penicillin G (BPG).^6^, ^7^ Timely screening and treatment of pregnant women with syphilis will lead to a significant reduction in these recognized adverse outcomes.^4^, ^5^ Findings from our team and others suggest that barriers to congenital syphilis elimination include ANC stockouts of test kits and BPG treatment.^8^, ^9^

Syphilis and HIV impact 1.4 million pregnant women globally and infection can be transmitted during pregnancy.^10^, ^11^, ^12^, ^13^ The prevalence of syphilis in pregnancy in Zambia ranges from 2% to 5% and HIV in pregnancy is approximately 10%.^14^, ^15^, ^16^, ^17^ Limited data suggest that pregnant women coinfected with syphilis and HIV have higher rates of adverse birth outcomes compared to pregnant women with syphilis alone.^18^ The objective of this study was to compare factors associated with adverse birth outcomes among pregnant women diagnosed with syphilis in the ANC clinic according to their HIV status.

## 2 MATERIALS AND METHODS

This retrospective cohort study was conducted among pregnant women who attended ANC clinics in 10 health facilities in urban Lusaka, Zambia, between January 1, 2018, and December 31, 2019. Birth outcomes were collected through June 30, 2020. The investigation was approved by the University of Zambia Biomedical Research Ethics Committee (no.: 1801‐2021) and the University of Alabama at Birmingham Institutional Review Board (IRB‐300007767). A waiver of informed consent was granted.

The selected 10 health facilities consisted of health clinics and first level hospitals that provided ANC services in Lusaka, Zambia. Each ANC clinic was staffed by nurses and counselors, with access to clinical officers and laboratory technicians, to provide comparable routine services for the diagnosis and treatment of syphilis. Data on pregnant women who attended ANC clinics at the selected 10 health facilities and were screened for syphilis between January 1, 2018, and December 31, 2019, were included in our analysis. According to the local standard of care at the time of the study, women were screened for syphilis with a qualitative rapid plasma reagin (RPR) test (Omega Diagnostics, UK) with no confirmatory antibody testing. Standard recommended syphilis treatment was BPG 2.4 million units administered intramuscularly every 7 days for three weeks.^19^ The study methodology has previously been published.^9^

Data were collected from government registers for women with positive syphilis screening during their ANC visit and subsequent delivery outcomes. We extracted demographic information and data on ANC attendance at hospital or health center facilities, documented BPG treatment rates, partner referral for therapy, location of delivery (facility vs. home), and birth outcomes. Adverse birth outcomes included low birth weight (<2500 g), preterm delivery (<37 weeks gestational age [GA]), stillbirth (≥28 weeks GA) or miscarriage (<28 weeks GA), congenital syphilis, neonatal mortality (≤28 days), and a composite measure that included any of the adverse outcomes listed.

### 2.1 Statistical analysis

Characteristics are reported as median and interquartile range (IQR) for continuous variables, and as number (proportion) for categorical variables. Comparison according to HIV status used Wilcoxon rank‐sum test or Pearson Chi‐square test/Fisher exact test, as appropriate. Simple logistic regression models were used to estimate the association between demographics, ANC information, syphilis information and the composite measure of adverse birth outcomes. Additional interactions between HIV status and these factors were evaluated. The final multivariable logistic regression model included variables that were significant, selected using backward elimination, to identify potential risk factors. A multivariable logistic regression model included variables with significant factors (*P* < 0.05) in simple models. Results are reported as odds ratios (ORs) with 95% confidence intervals (CIs). All analyses were performed using SAS (version 9.4; SAS Institute; Cary, NC), with level of significance set at *P* values less than 0.05 for all analyses. There was no imputation for missing data.

## 3 RESULTS

A total of 1204 pregnant women who screened positive for syphilis were identified during ANC care. The median maternal age was 27, and 19.8% of women were <14 weeks' gestation at the time of ANC entry to care (Table 1). Among the 512 (42.5%) pregnant women with HIV, 161 (31.4%) had been newly diagnosed with HIV during ANC care. Most women (51.4%) had parity ranging from 1 to 2, with very little difference (50.6% and 52.4%) by HIV status. Only 48.1% (579/1204) of women had penicillin treatment documented, and treatment rates were similar when stratified by HIV. Referral of sex partners for syphilis therapy was infrequent and slightly higher among women with HIV (9.8% vs. 6.1% in women without HIV; *P* = 0.02) (Table 1). Syphilis screening and treatment rates did not differ by HIV status.

**TABLE 1:** Characteristics of pregnant women with syphilis according to HIV status.

Characteristic	Women without HIV Median (IQR) or *n* (%) (*n* = 692)	Women with HIV Median (IQR) or *n* (%) (*n* = 512)	Total Median (IQR) or *n* (%) (*n* = 1204)	*P* value
Demographics				
Age (years) (*n* = 1192)	26.0 (22.0, 31.0)	28.0 (25.0, 34.0)	27.0 (23.0, 32.0)	<0.001
Age category (years)				<0.001
<20	53 (7.7)	13 (2.6)	66 (5.5)	
20–24	215 (31.2)	96 (19.1)	311 (26.1)	
25–29	206 (29.9)	168 (33.4)	374 (31.4)	
30–34	118 (17.1)	118 (23.5)	236 (19.8)	
35–39	75 (10.9)	86 (17.1)	161 (13.5)	
40+	22 (3.2)	22 (4.4)	44 (3.7)	
Married (*n* = 1132)	577 (87.7)	421 (88.2)	998 (88.2)	0.562
Parity (*n* = 1176)				0.059
0	157 (23.2)	86 (17.3)	243 (20.7)	
1–2	343 (50.6)	261 (52.4)	604 (51.4)	
3–4	148 (21.8)	120 (24.1)	268 (22.8)	
5+	30 (4.4)	31 (6.2)	61 (5.2)	
ANC information				
Facility type (*n* = 1204)				0.410
Hospital	403 (58.2)	286 (55.9)	689 (57.2)	
Health center	289 (41.8)	226 (44.1)	515 (42.8)	
Gestational age at entry to ANC (*n* = 1076)				0.127
<14 weeks	121 (19.4)	92 (20.4)	213 (19.8)	
14–27 weeks	461 (73.9)	315 (69.7)	776 (72.1)	
28+ weeks	42 (6.7)	45 (9.9)	87 (8.1)	
HIV status (1204)				
Negative	692 (100)	0 (0)	692 (57.5)	
Prevalent (known positive)	0 (0)	351 (68.6)	351 (29.1)	
Incident (new diagnosis)	0 (0)	161 (31.4)	161 (13.4)	
Documented syphilis treatment (*n* = 1204)	341 (49.3)	238 (46.5)	579 (48.1)	0.338
Time between syphilis diagnosis and treatment (*n* = 557)				0.103
0 days	270 (81.6)	185 (81.9)	455 (81.7)	
1–7 days	47 (14.2)	28 (12.4)	75 (13.5)	
8–30 days	6 (1.8)	11 (4.9)	17 (3.0)	
>30 days	8 (2.4)	2 (0.9)	10 (1.8)	
Partner referral for syphilis (*n* = 1204)	68 (9.8)	31 (6.1)	99 (8.2)	0.019

Abbreviations: ANC, antenatal care; IQR, interquartile range.

Delivery outcomes were available for 813/1204 women in the ANC cohort (Table 2). Facility delivery rates were high in both groups (97.2% in women with HIV vs. 94.5% in women without HIV; *P* = 0.05). The only outcome that differed by HIV status was preterm delivery with higher rates in women with HIV (39.9% vs. 30.0% in women without HIV; *P* = 0.003). Any adverse birth outcome occurred in 43.6% in women with HIV and 40.7% in women without HIV (*P* = 0.398).

**TABLE 2:** Birth and neonatal outcomes in pregnant women with syphilis by HIV status (*n* = 813).

Variable	Women without HIV Median (IQR) or *n* (%) (*n* = 467)	Women with HIV Median (IQR) or *n* (%) (*n* = 346)	Total Median (IQR) or *n* (%) (*n* = 813)	*P* value
Facility delivery	454 (97.2%)	327 (94.5%)	781 (96.1%)	0.050
Birth outcome				0.530^a^
Live birth	460 (98.5%)	343 (99.1%)	803 (98.8%)	
Stillbirth (≥28 weeks)	7 (1.5%)	3 (0.9%)	10 (1.2%)	
Miscarriage (<28 weeks)	8 (1.7%)	6 (1.7%)	14 (1.7%)	
Sex (female)	227 (48.6%)	155 (44.8%)	382 (47.0%)	0.282
Twin delivery	9 (1.9%)	5 (1.5%)	14 (1.7%)	0.601
Birth weight (g)^b^	3000 (2600, 3300)	3000 (2610, 3200)	3000 (2610, 3200)	0.168
Low birth weight (<2500 g)	61 (13.1%)	36 (10.4%)	97 (11.9%)	0.248
Preterm delivery (<37 weeks)	147 (30.0%)	129 (39.9%)	276 (34.0%)	0.003
Neonatal mortality^c^	5 (1.1%)	5 (1.5%)	10 (1.2%)	0.751^a^
Congenital syphilis	2 (0.4%)	0	2 (0.3%)	0.510^a^
Adverse birth outcome^d^	190 (40.7%)	151 (43.6%)	341 (41.9%)	0.398

Abbreviation: IQR, interquartile range.

^a^ Indicates Fisher exact test.

^b^ Indicates birth weight among live singletons.

^c^ Neonatal mortality (≤28 days) is based on available records and may be incomplete.

^d^ Includes low birth weight, preterm birth, stillbirth or miscarriage, neonatal death, and congenital syphilis.

Two factors were associated with adverse birth outcomes in unadjusted models: earlier entry to care (<14 weeks GA) and receiving ANC at a health center compared to a hospital facility (Table 3). After adjustment, pregnant women who received care at a health facility (OR: 1.39; 95% CI: 1.03–1.87) and pregnant women with lack of documented syphilis treatment had higher odds of having an adverse birth outcomes (OR: 1.44; 95% CI: 1.07–1.93), while those with 14–27 weeks (OR: 0.51; 95% CI: 0.35–0.74) and for >28 weeks GA at ANC entry to care (OR: 0.34; 95% CI: 0.17–0.66) had lower odds of an adverse birth outcome. Maternal age, parity, HIV status, and delay in treatment were not associated with outcomes.

**TABLE 3:** Factors associated with adverse birth outcomes in pregnant women with syphilis.

	OR (95% CI)	*P* value	Adjusted OR (95% CI)	*P* value
Age (*n* = 809)		0.742		
16–24	0.95 (0.71, 1.28)			
25+	REF			
Parity (*n* = 800)		0.269		
0	REF			
1–2	1.41 (0.97, 2.05)			
3–4	1.15 (0.75, 1.78)			
5+	1.08 (0.54, 2.18)			
Gestational age at ANC entry (*n* = 742)		<0.001		<0.001
<14 weeks	REF		REF	
14–27 weeks	0.48 (0.33, 0.70)		0.52 (0.36, 0.75)	
28+ weeks	0.31 (0.16, 0.59)		0.35 (0.18, 0.67)	
ANC facility type (*n* = 813)		0.024		0.032
Hospital	REF		REF	
Health center	1.39 (1.04, 1.84)		1.39 (1.03, 1.87)	
HIV status (*n* = 813)		0.398		
Negative	REF			
Positive	1.13 (0.85, 1, 50)			
No documented syphilis treatment (*n* = 813)	1.30 (0.98, 1.72)	0.068	1.44 (1.07, 1.93)	0.015
Time between syphilis diagnosis and treatment (*n* = 376)		0.577		
0 day	REF			
1–7 days	1.36 (0.77, 2.40)			
8+ days	1.03 (0.39, 2.73)			

Abbreviations: ANC, antenatal care; CI, confidence interval; OR, odds ratio.

Furthermore, we used bivariate analyses to identify potential risk factors for adverse outcomes and tested interactions to determine whether the effects of these risk factors varied by HIV status. Although we observed ORs different from one, only three were statistically significant, and none of the interactions were significant. Therefore, the final model did not include HIV (Appendix S1).

## 4 DISCUSSION

In this study, we found that similarly elevated rates of adverse birth outcomes occurred among women in Zambia who screened positive for syphilis during ANC care. Women with HIV and syphilis coinfection had a higher preterm birth rate compared to pregnant women with syphilis alone. This finding is consistent with other literature from Botswana, Brazil, and Lesotho.^18^, ^20^, ^21^ Other studies from Botswana, the Democratic Republic of Congo, and Lesotho have found an association between HIV status and other adverse outcomes (stillbirths, miscarriage, and neonatal deaths), which our study did not demonstrate.^18^, ^20^, ^21^, ^22^, ^23^ Since some of these studies were older, some of the differences could be attributed to improved HIV care and outcomes in SSA over time.

Given the well‐described association between untreated syphilis in pregnancy and adverse outcomes in the current study and many others, it is of concern that only 48% of pregnant women diagnosed with syphilis in our study had documented penicillin treatment.^13^, ^18^, ^22^

A prospective cohort study conducted in Ethiopia found that parity or gravida was not a risk factor for increased adverse birth outcomes in women with HIV, which aligns with our study findings.^24^, ^25^ Access to skilled care during facility deliveries prevents deaths and is also beneficial for the prevention of maternal‐to‐child HIV transmission (PMTCT).^26^ Facility delivery was nearly universal in our cohort, with higher rates in women with HIV. This confirms other published data from Zambia.^27^

We were surprised to see in our model that women who sought ANC during their second and third trimesters had fewer adverse birth outcomes compared to those who sought early ANC care. In Ethiopia and Ghana, first trimester initiation of ANC was associated with reduced rates of adverse birth outcomes.^28^, ^29^, ^30^ Some studies highlight that early ANC care does not always correlate with adequate ANC follow‐up care and lack of follow‐up has been associated with adverse outcomes.^31^, ^32^ In the current study, we found that women who sought ANC care at the health facility had a higher chance of an adverse birth outcome as compared to those attending ANC at hospital facilities, which may reflect variations in quality of ANC services available for women. A multinational trial found high rates of inadequate services throughout the healthcare system in many developing countries, which likely contribute to high rates of adverse birth outcomes.^33^

Despite advances in early identification and treatment of pregnant women with syphilis, maternal reinfection and mother‐to‐child transmission remain a critical problem due to untreated male partners.^34^ A case–control study in China found that pregnant women with an untreated male partner with syphilis are at a greater risk of having adverse birth outcomes, including congenital syphilis.^35^ Therefore, partner referral for sexually transmitted infections (STI) services is a logical step to reduce STI reinfection in pregnancy, but it can be challenging to put into practice.^36^, ^37^, ^38^ Similar to our study with a partner referral rate of 8%, a recent randomized controlled trial conducted in Uganda found that 12% of male partners of pregnant women with syphilis attended the health facility for testing as recommended.^19^ Among partners who tested positive, only 18% received treatment.^19^

Our study had the following strengths and limitations. We were not able to confirm the RPR results with treponemal testing, therefore, we may have overestimated the rate of active syphilis infection in our cohort. We were missing data on all delivery outcomes due to the limitations of paper‐based government health records, which limit the interpretation of some findings. We assumed the data were missing at random. Nevertheless, we have interpreted our findings with appropriate caution. Lastly, our study findings may not be generalizable to pregnant women who do not seek ANC care. However, the sample size of women seeking ANC care in a variety of facilities in a setting where HIV and syphilis are prevalent contributes to the strength of this study.

## 5 CONCLUSION

Among pregnant women diagnosed with syphilis in Zambia, treatment rates were low and adverse birth outcomes were high. Although most outcomes did not differ by HIV status, preterm birth rates were higher in women with HIV and syphilis compared to syphilis alone.

## AUTHOR CONTRIBUTIONS

Albert Manasyan was responsible for the study implementation, data collection, and drafting of this manuscript. Anna V. Jones contributed to drafting the manuscript and provided suggestions. Yumo Xue conducted the statistical analysis and reviewed the manuscript. Herbert Kapesa conducted the data clean‐up and reviewed the manuscript. Maureen Mzumara reviewed the manuscript. Jodie A. Dionne developed the study protocol, interpreted the study findings, and reviewed the manuscript. Mwangelwa Mubiana‐Mbewe contributed to the study development and reviewed the manuscript.

## FUNDING INFORMATION

No funding was received for this research.

## CONFLICT OF INTEREST STATEMENT

The authors report no conflicts of interest.

## Supporting information


**Appendix S1.** Bivariate analyses for identification of potential risk factors for adverse outcomes by HIV status.

## Data Availability

The data that support the findings of this study are available from the corresponding author upon reasonable request.
