# Supplementary material for: Low syphilis treatment rates and associated birth outcomes in pregnant women with and without HIV in Zambia: A cohort study
Source: Int J Gynaecol Obstet. 2025 Sep 3;172(3):1569–75. doi: 10.1002/ijgo.70518 (PMC12936629; doi:10.1002/ijgo.70518)
Supplement: Supplementary file 1 — Appendix S1. Bivariate analyses for identification of potential risk factors for adverse outcomes by HIV status. [file IJGO-172-1569-s001.docx]

**Supplementary Appendix S1:** Bivariate analyses for identification of potential risk factors for adverse outcomes by HIV status.

|  | **Odds Ratio (OR) (95% CI)**  **No HIV** | **Odds Ratio (OR) (95% CI)**  **With HIV** |
| --- | --- | --- |
| **Age (n=809)**  16-24  25+ | 0.865 (0.598,1.252)  REF | 1.141 (0.697,1.868)  REF |
| **Parity (n=800)**  0  1-2  3-4  5+ | REF  1.966 (1.207,3.202)  1.580 (0.890,2.805)  1.850 (0.668,5.119) | REF  0.818 (0.452,1.480)  0.681 (0.344,1.348)  0.533 (0.196,1.451) |
| **Gestational Age at ANC Entry (n=742)**  <14 weeks  14-27 weeks  28+ weeks | REF  0.557 (0.340,0.911)  0.286 (0.109,0.753) | REF  0.413 (0.236,0.723)  0.309 (0.124,0.770) |
| **ANC Facility Type (n=813)**  Hospital  Health Center | REF  1.39 (1.04,1.84) | REF  1.35 (0.99,1.83) |
| **No Documented Syphilis Treatment (n=813)** | 1.533 (1.057,2.224) | 1.034 (0.675,1.582) |
| **Time between Syphilis Diagnosis and Treatment (n=376)**  0 day  1-7 days  8+ days | REF  1.484 (0.718,3.068)  0.795 (0.199,3.183) | REF  1.218 (0.482,3.078)  1.340 (0.320,5.603) |
